# High Intensity Training May Reverse the Fiber Type Specific Decline in Myogenic Stem Cells in Multiple Sclerosis Patients

**DOI:** 10.3389/fphys.2016.00193

**Published:** 2016-05-31

**Authors:** Jean Farup, Ulrik Dalgas, Charly Keytsman, Bert O. Eijnde, Inez Wens

**Affiliations:** ^1^Section of Sport Science, Department of Public Health, Aarhus UniversityAarhus, Denmark; ^2^Research Laboratory for Biochemical Pathology, Department of Clinical Medicine, Aarhus UniversityAarhus, Denmark; ^3^Faculty of Medicine and Life Sciences, REVAL Rehabilitation Research Center, BIOMED Biomedical Research Institute, Hasselt UniversityDiepenbeek, Belgium

**Keywords:** satellite cells, myonuclei, skeletal muscle atrophy, multiple sclerosis, fibrosis

## Abstract

Multiple sclerosis (MS) is associated with loss of skeletal muscle mass and function. The myogenic stem cells (satellite cells—SCs) are instrumental to accretion of myonuclei, but remain to be investigated in MS. The present study aimed to compare the SC and myonuclei content between MS patients (*n* = 23) and age matched healthy controls (HC, *n* = 18). Furthermore, the effects of 12 weeks of high intensity training on SC and myonuclei content were explored in MS. Muscle biopsies were obtained from m. Vastus Lateralis at baseline (MS and HC) and following 12 weeks of training (MS only). Frozen biopsies were sectioned followed by immunohistochemical analysis for fiber type specific SCs (Pax7^+^), myonuclei (MN) and central nuclei content and fiber cross-sectional area (fCSA) was quantified using ATPase histochemistry. At baseline the SCs per fiber was lower in type II compared to type I fibers in both MS (119%, *p* < 0.01) and HC (69%, *p* < 0.05), whereas the SCs per fCSA was lower in type II fibers compared to type I only in MS (72%, *p* < 0.05). No differences were observed in MN or central nuclei between MS and HC. Following training the type II fiber SCs per fiber and per fCSA in MS patients increased by 165% (*p* < 0.05) and 135% (*p* < 0.05), respectively. Furthermore, the type II fiber MN content tended (*p* = 0.06) to be increased by 35% following training. In conclusion, the SC content is lower in type II compared to type I fibers in both MS and HC. Furthermore, high intensity training was observed to selectively increase the SC and myonuclei content in type II fibers in MS patients.

## Introduction

Multiple sclerosis (MS) is a chronic disease of the central nervous system that is often accompanied by a sedentary lifestyle (Miller and Dishon, 2006). This affects skeletal muscle characteristics in these patients, as evidenced by reduced skeletal muscle fiber cross-sectional area (fCSA) and muscle mass of the lower limbs, leading to reduced muscle strength (Wens et al., 2014). In turn the reduced muscle strength affects functional capacity and subsequently quality of life (Compston and Coles, 2002). Consequently, a thorough understanding of muscle biology is important to optimize the preservation of muscle function and thereby functional capacity in MS.

In this regard, an essential aspect of muscle repair, growth, and remodeling is a functional population of quiescent muscle specific stem cells, commonly referred to as satellite cells (SCs) (Brack and Rando, 2012; Montarras et al., 2013; Yin et al., 2013). Since the discovery and proposed role as a resident pool of quiescent myogenic stem cells (Mauro, 1961), the SCs have been shown to possess a critical role in animal skeletal muscle regeneration (Lepper et al., 2011; von Maltzahn et al., 2013), during which SCs are activated, proliferate, and differentiate to support regeneration and new myofiber formation.

While the prerequisite for SCs during the initial phase of myofiber growth is currently controversial (Adams et al., 2002; Petrella et al., 2008; McCarthy et al., 2011; Sambasivan et al., 2011; Guerci et al., 2012; Blaauw and Reggiani, 2014; Biressi and Gopinath, 2015) the SCs become important for continued myofiber growth at later stages (Fry et al., 2014a). These findings in rodents are supported by a number of studies in healthy human skeletal muscle reporting that the SC activity and content increases following acute (i.e., hours-days) resistance exercise and the SC content remains elevated following prolonged (i.e., weeks-months) resistance training (Kadi et al., 2004; Olsen et al., 2006; Petrella et al., 2008; Verdijk et al., 2009a; Mackey et al., 2010, 2011; McKay et al., 2012; Snijders et al., 2012, 2013). Interestingly, conditional ablation of SCs in both young and old (sarcopenic) mice is associated with increased fibroblast proliferation (Tcf4^+^ cells) and connective tissue content (fibrosis) (Fry et al., 2014a, 2015). In addition, co-culture experiments with primary cells from mice skeletal muscle suggest that factors released from SCs may also regulate adipogenic differentiation of fibro-adipogenic progenitors during muscle regeneration (Uezumi et al., 2010). Collectively, SC content and function may be important for muscle hypertrophy as well as controlling and ensuring a healthy muscle microenvironment (changes in fibrotic and adipocyte content).

Both aging and disease may impair the capacity for SCs to support myofiber regeneration (Conboy et al., 2005; Chakkalakal et al., 2012; Cosgrove et al., 2014; Price et al., 2014), which is ascribed to alterations in the SC niche or the systemic environment (Conboy et al., 2005; Carlson et al., 2009; Chakkalakal et al., 2012; Elabd et al., 2014; Sinha et al., 2014) negatively influencing SC proliferation and differentiation. In rodents, sarcopenia is not accelerated when SCs are ablated (approximately 90% efficiency of SC ablation), however, loss of SCs is associated with increased fibrosis (Fry et al., 2015), which may compromise muscle function. In humans, aging is associated with a decrease in type II fiber size (Kryger and Andersen, 2007; Verdijk et al., 2009a; Suetta et al., 2013) and a preferential reduction in SCs associated with type II fibers (Verdijk et al., 2009a, 2013; Mackey et al., 2013). Moreover, the aging muscle microenvironment is associated with increased fibrosis and adipocyte infiltration (Conley et al., 1995; Mann et al., 2011). In contrast to aging, there is sparse knowledge concerning the SC content in humans suffering from chronic diseases. Studies in type II diabetes (Snijders et al., 2011), chronic obstructive pulmonary disorders (Theriault et al., 2014), rheumatoid arthritis (Beenakker et al., 2012) or patients undergoing dialysis (Molsted et al., 2015) does, however, not indicate a lower SC content in these conditions, although further research is necessary. So far, no studies have evaluated if a chronic neurological condition, such as MS, affects SC content and the ability of the SCs to proliferate and differentiate. Such knowledge is important in order to identify and understand mechanisms underlying muscle atrophy and dysfunction and to develop strategies to preserve functional capacity and avoid muscle deterioration.

Resistance exercise has attracted much attention as it is known to increase the fCSA of the type II fibers in healthy subjects (Folland and Williams, 2007; Verdijk et al., 2009a; Farup et al., 2012) as well as in MS patients (Dalgas et al., 2010). Following resistance exercise there is an increased proliferation of SCs, specifically associated with type II fibers, in young, and elderly individuals (Snijders et al., 2009; McKay et al., 2012). Moreover, the type II fiber SC content remains increased in elderly following prolonged resistance training (Verdijk et al., 2009a). In addition to resistance exercise alone (Dalgas et al., 2010) the combination of endurance and resistance exercise improves various aspects of the physiological profile of MS patients and may constitute the optimal exercise stimulus in MS (Doring et al., 2011; Wens et al., 2015). Of note, endurance exercise may also stimulate SC proliferation (Joanisse et al., 2013; Fry et al., 2014b) although it is not yet clear if this proliferation is fiber type specific. Taken together, fundamental questions related to the effects of combined endurance and resistance exercise on SC still needs to be addressed in pathological conditions including MS.

This study set out to compare the SC content and the muscle environment in MS patients and healthy matched controls as well as to evaluate the effect of a 12-week training program consisting of combined endurance and resistance training on SC content in MS patients. It was hypothesized that the type II fiber SC and myonuclei content was reduced in MS patients compared to healthy controls and that the 12-week training program could reverse this decline in SC and myonuclei content in type II fibers. Furthermore, we hypothesized that MS patient's skeletal muscle would display an increased level of fibrosis and lipid content [reflective of intramyocellular as well as intermyocellular (e.g., adipocytes) lipid content] compared to healthy age and activity matched controls.

## Materials and methods

### Participants

Twenty-three MS patients diagnosed according to McDonald criteria (Expanded disability status scale; range 1–6) and 18 matched healthy controls (HC), aged >18 years, were included following written informed consent. Subjects were excluded if they had other disorders (cancer, cardiovascular, pulmonary, and/or renal), were pregnant, participated in another study, had an acute MS-exacerbation 6 months prior to the start of the study or contra-indications to perform physical exercise. The study was approved by the Ethical committee at Hasselt University and by the Ethical committee at Jessa Hospital in Hasselt, and the study was registered at ClinicalTrials.gov (NCT01845896). All study-procedures followed the Declaration of Helsinki.

### Study design overview

At the start of the study knee flexor and extensor strength, body composition, maximal endurance capacity, and self-reported physical activity levels were assessed in MS patients (Wens et al., 2014). Next, (separated by at least 48 h) m. Vastus Lateralis muscle biopsies were collected from MS patients and HC. Subjects were then randomized into one of two exercise groups performing 12 weeks of a high intensity interval + resistance training (H_IT_R, *n* = 12) or high intensity continuous endurance + resistance training (H_CT_R, *n* = 11), respectively. Neither the patients nor the researchers involved in the project were blinded to group allocation. All testing was repeated after 12 weeks.

### Exercise intervention program

After baseline measurements, the subjects were enrolled in a well-controlled and supervised training program, to increase cardiorespiratory fitness, as well as strength of the major peripheral muscle groups. Subjects participated in five sessions per 2 weeks. Training sessions were interspersed by at least 1 day of rest, to ensure adequate recovery.

#### H_IT_R program

High intensity cycle interval training was performed during the first 6 weeks where exercise duration gradually increased from 5 × 1 min interspersed by 1 min rest intervals to 5 × 2 min and 1 min rest intervals. Exercise intensity was defined as the heart rate, corresponding to 100% of the maximal workload obtained during the initial incremental test. During the second 6 weeks, the exercise duration remained stable at 5 × 2 min and the workload increased to reach a level corresponding to 100–120% of the maximal workload. The second part consisted of moderate-to-high intensity resistance training (leg press, leg curl, leg extension, vertical traction, arm curl, and chest press). In order to exercise at similar relative workload, resistance training of the lower limb was performed unilaterally, due to the frequent bilateral strength differences seen between the legs of persons with MS (Thoumie et al., 2005). Training intensity and volume were adjusted from 1 × 10 repetitions to 2 × 20 repetitions at maximal attainable load.

#### H_CT_R program

Session duration and exercise intensity increased as the intervention progressed, starting from 1 × 6 min/session to 2 × 10 min/session, at a high workload, corresponding to 80–90% of maximal heart rate and according to individual capabilities. The second part of the training session comprised similar resistance training, as described in the H_IT_R program.

### Outcome measures

#### Biopsy collection and preparation

To investigate muscle fiber SC, myonuclei, central nuclei content as well as fiber CSA, muscle biopsies form the middle part of the m. Vastus Lateralis (Bergström needle technique) of the weakest leg (see isometric muscle strength measurements) were collected by an experienced medical doctor. The post-intervention biopsy was taken 2–3 cm proximal to the biopsy taken at baseline. Muscle samples were immediately mounted with Tissue-Tek, frozen in isopentane cooled with liquid nitrogen and stored at −80°C, until further analysis. The cross-sections of the biopsies, collected at baseline and after 12 weeks, were processed simultaneously.

#### Satellite cell, myonuclei, and central nuclei analysis

Muscle biopsy sections were fixed in Histofix (Histolab, Gothenborg, Sweden) followed by 1.5 h in blocking buffer (0.2% Triton-X, 2% BSA, 5% FBS, 2% goat serum, and 0.1% sodium azide). The sections were incubated overnight at 4°C with primary antibody for Pax7 (1:500; cat. no MO15020, Neuromics, Edina, MN, USA), followed by 1.5 h in secondary Alexa Fluor 568 goat anti-mouse antibody (1:200; Molecular Probes, cat no. A11034, Invitrogen A/S, Taastrup, Denmark). Following this, the sections were incubated with primary antibodies for Type I myosin [1:500; cat. no. A4.951, Developmental Studies Hybridoma Bank (DSHB), IA, USA] and laminin (1:500; cat. no. Z0097, Dako Norden) for 2 h and secondary Alexa Fluor 488 goat anti-mouse green and Alexa Fluor 488 goat anti-rabbit green (1:500; Molecular Probes, cat no. A11031 and cat no. A11034, Invitrogen A/S, Taastrup, Denmark) antibodies for 1 h. Finally, a mounting media containing 4′,6-Diamidino-2-phenylindole (DAPI) was utilized to visualize nuclei (Molecular Probes Prolog Gold anti-fade reagent, cat. no. P36935, Invitrogen A/S) and samples were stored at −20°C until final analyses. Staining was verified using appropriate negative controls to ensure specificity.

Images were obtained at 20x magnification using a Leica DM2000 microscope (Leica, Stockholm, Sweden) and a Leica Hi-resolution Color DFC camera (Leica, Stockholm, Sweden). The number of Pax7 positive (Pax7^+^) cells (SCs) associated with type I (A4.951^+^) or type II (A4.951^−^) fibers was quantified separately and expressed relative to the total number of type I or II fibers and fiber area (SC/mm^2^). To ensure reliable numbers of SCs, in accordance with Mackey et al. (2009), we counted a mean of 246 ± 3 fibers on each biopsy.

Finally, in accordance with Bruusgaard et al. (2012), we utilized these sections to quantify total sublaminar nuclei (assumed to be myonuclei) by only counting Pax7 negative nuclei with a visible geometric center within the basal lamina to ensure that non-myonuclei were not counted. When located centrally in the myofiber the nuclei were enumerated as a central nuclei. The myonuclei and central nuclei content from total type I and type II fibers were enumerated and normalized to the fiber number.

Initially two individuals performed the SC and myonuclei quantifications on the same sections to ensure appropriate quantifications. Following this, one individual, who was blinded for subject identity, performed all SC, and myonuclei/central nuclei quantifications.

#### Muscle fiber CSA and proportion

The fiber type specific CSA (fCSA) differences between MS and HC as well as before and after training have been reported previously (Wens et al., 2014, 2015). In brief, mean fCSA, as well as fCSA of type I, II, and IIa fibers were approximately 18% smaller in MS patients, compared to HC. Interestingly, 12 weeks of exercise was able to reverse mean fCSA, as well as fCSA of type I, II, and IIa fibers (Wens et al., 2014, 2015).

In the present paper we utilized the fiber CSA from type I and type II fibers as a second normalization strategy to account for the potential influence of fiber size on the SC content. To briefly describe the fiber area measurements, serial transverse sections (9 μm) from the obtained muscle samples were cut at −20°C and stained by means of ATPase histochemistry, after preincubation at pH 4.4, 4.6, and 10.3, essentially following the procedure of Brooke and Kaiser (Brooke and Kaiser, 1970). The serial sections were visualized and analyzed using a Leica DM2000 microscope (Leica, Stockholm, Sweden) and a Leica Hi-resolution Color DFC camera (Leica, Stockholm, Sweden) combined with image-analysis software (Leica Qwin ver. 3, Leica, Stockholm, Sweden). A fiber mask of the stained sections was drawn automatically and manually fitted to the cell borders of the selected fibers. Only fibers cut perpendicularly to their longitudinal axis were used for the determination of fiber size. Calculation of the fiber CSA for the SC normalizations was performed only for type I and type II (IIa+IIx) muscle fiber phenotypes. SCs in type I and II fibers were normalized to type I and II fiber CSA, respectively [SCs × (mm^2^ fCSA)^−1^)]. Additionally, the myonuclear domain size (μm^2^ fCSA × myonuclei^−1^) was calculated for type I and II fibers.

### Muscle tissue fibrosis and lipid content

To evaluate muscle fibrotic tissue content we quantified *N*-acetyl-d-glucosamine content in accordance with previous studies (Fry et al., 2014a, 2015). Sections were fixed 4 min in Histofix and incubated for 1 h with Alexa Fluor 488 conjugated Wheat Germ Agglutinin (5 μg/ml, cat no. W11261, Invitrogen A/S). For lipid quantification we stained the section in accordance with previously published protocols (Mehlem et al., 2013). In brief, Oil-Red-O (Direct Red 80, lot# MKBP5683V, Sigma-Aldrich, Copenhagen, DK) working solution was prepared from 15 ml of stock solution (2.5 g Oil-Red-O, 400 ml 99% isopropyl) and 10 ml water, left to fridge for 10 min at 4°C, and filtered through 45-μm filter. The muscle sections were stained for 10 min in the working solution rinsed for 40 min in running tap-water and mounted in pertex medium (Pertex, lot# 03649935, CellPath, UK).

For quantification of fibrosis and lipids, five random images from each biopsy were collected and analyzed using the threshold and area quantification (in mm^2^) function in ImageJ. To account for changes in fiber size, for the fibrosis stain, the area was normalized to fiber number. Due to lack of sufficient sample quality in some biopsies the number of included samples in this analysis was somewhat smaller than for the SC analysis (*n* = 9–16 per group/time-point).

### Statistical analysis

Following check for normality using qq-plots and histogram inspection and tests of equal variance (Bartletts test) the data were expressed as mean ± SEM in text or as individual values with mean bars and SEM in figures. The central nuclei data were displayed as individual plots and median bars. Baseline comparison between MS and HC were performed using unpaired *t*-test or Wilcoxon-Mann-Whitney test (only for central nuclei). Differences in SCs, myonuclei, myonuclear domain between type I and II fibers were conducted using a paired *t*-test while a Wilcoxon-Mann-Whitney test was utilized for central nuclei.

The effect of time (*Pre* vs. *Post*) and group (*H*_*IT*_*R* vs. *H*_*CT*_*R*) and their interaction on dependent variables was assessed using a mixed-effect linear model with repeated measures for time using subject id as a random effect variable. This analysis revealed no effect of group (*H*_*IT*_*R* vs. *H*_*CT*_*R*) on any of the dependent variables. Since no effects were observed and to enhance the clarity of the paper and the power of the data the two training groups were merged and only the effect of time was examined (i.e., overall effect of training in general). As described in the section above the central nuclei data showed a non-parametric distribution and to examine the effect of training in general we performed a Wilcoxon-Mann-Whitney test on the two training groups collapsed. To describe the association between SC content, myonuclei, and single fCSA, a Pearson product-moment correlation analysis was employed on pre- (MS and HC combined) and post-(MS only) training data separately. The alpha level was set to *p* ≤ 0.05. All statistical analyses were performed using Stata (Stata v 12.0, StataCorp LP, TX, USA).

## Results

### Satellite cells, myonuclei, and central nuclei; MS vs. HC

**Figure 1:**
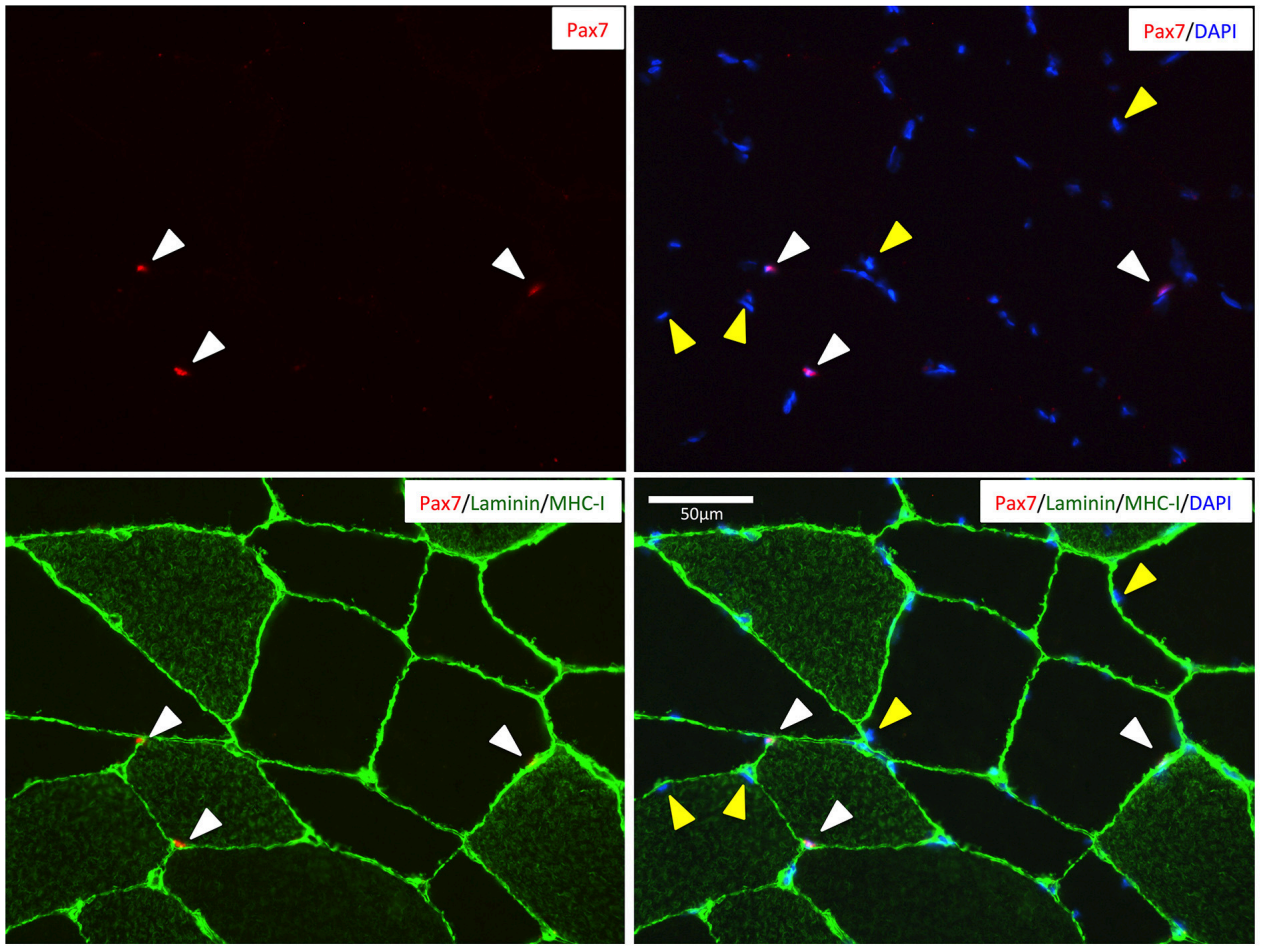
**Representative image of a muscle cross-section stained for Pax7 (red), MCH-I (green), laminin (green), and nuclei (DAPI; blue)**. White cones indicates the location of the Pax7^+^ satellite cells beneth the basal lamina of two type II fibers (MHC^−^) and one type I fiber (MHC^+^). Yellow cones indicate nuclei identified as myonuclei (i.e., Pax7^−^ nuclei beneath the basal lamina). Scale bar is 50 μm.

A representative image of Pax7^+^ cells localized beneath the basal lamina (i.e., SCs) of type I (MHC-I^+^) or type II (MHC-I^−^) fibers as well as sublaminar Pax7^−^ nuclei (i.e., myonuclei) is provided in Figure 1.

**Figure 2:**
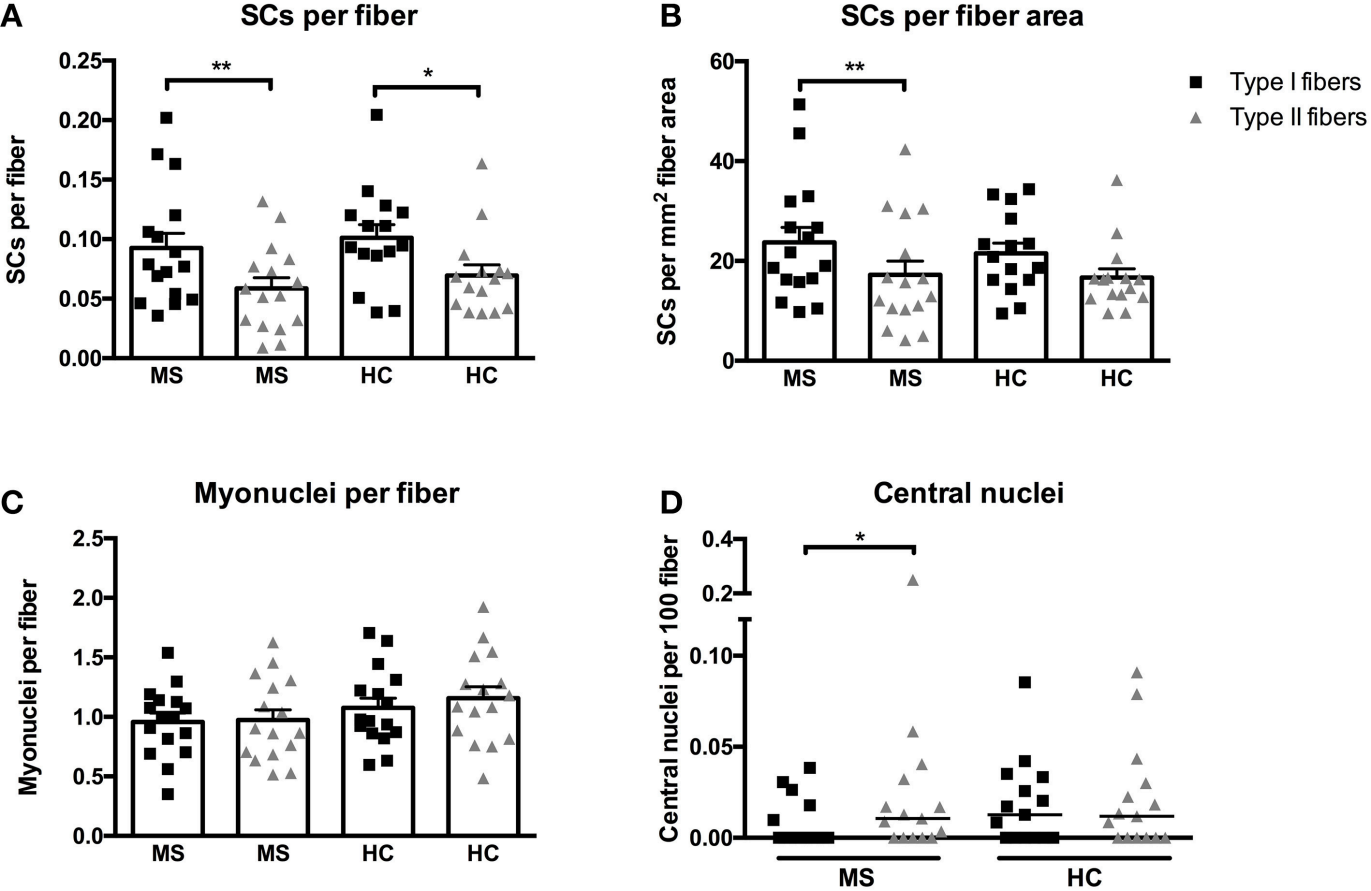
**Satellite cells (SCs) associated with type I or II fibers expressed per fiber (A) or per mm^**2**^ fiber area (B), myonuclei per fiber (C), and central nuclei per fiber (D) evaluated from biopsies obtained from patients with multiple sclerosis (MS) and from healthy controls (HC)**. Data are presented as individual values and mean ± SEM **(A–C)** or individual data-points and median bar **(D)**. Significant differences between type I and type II fibers are denoted by ^**^p < 0.05 or ^**^p < 0.01.

At baseline the SCs per fiber and SCs per mm^2^ fiber area in type I as well as type II fibers was similar between MS patients and HC (Figures 2A,B). When expressed as SCs per fiber the SC content of type II fibers was 119 ± 39% (*p* < 0.01) and 69 ± 21% (*p* < 0.05) lower compared to type I fibers, in MS and HC, respectively. Conversely, when expressed as SCs per mm^2^ fiber area only MS displayed a 72 ± 21% (*p* < 0.05) lower SC content in type II fibers compared to type I fibers.

As for myonuclei no significant differences were observed between MS and HC, (*p* = 0.15, Figure 2C). In contrast to SCs no differences in myonuclear content were observed between fiber types in neither MS nor HC. The myonuclear domain (fCSA × myonuclei^−1^) was greater in type I (4568 ± 244 μm^2^ × myonuclei^−1^) as compared to type II fibers (3829 ± 229 μm^2^ × myonuclei^−1^) in MS and HC combined (*p* < 0.01), whereas no group differences were observed.

The content of central nuclei (an indicator of myofiber regeneration/remodeling) in type I or II fibers was not different between MS and HC (Figure 2D). However, a difference in central nuclei content between type I and II fibers was observed in MS (*p* < 0.05) but not in HC.

### Satellite cells, myonuclei, and central nuclei; effect of training in MS

**Figure 3:**
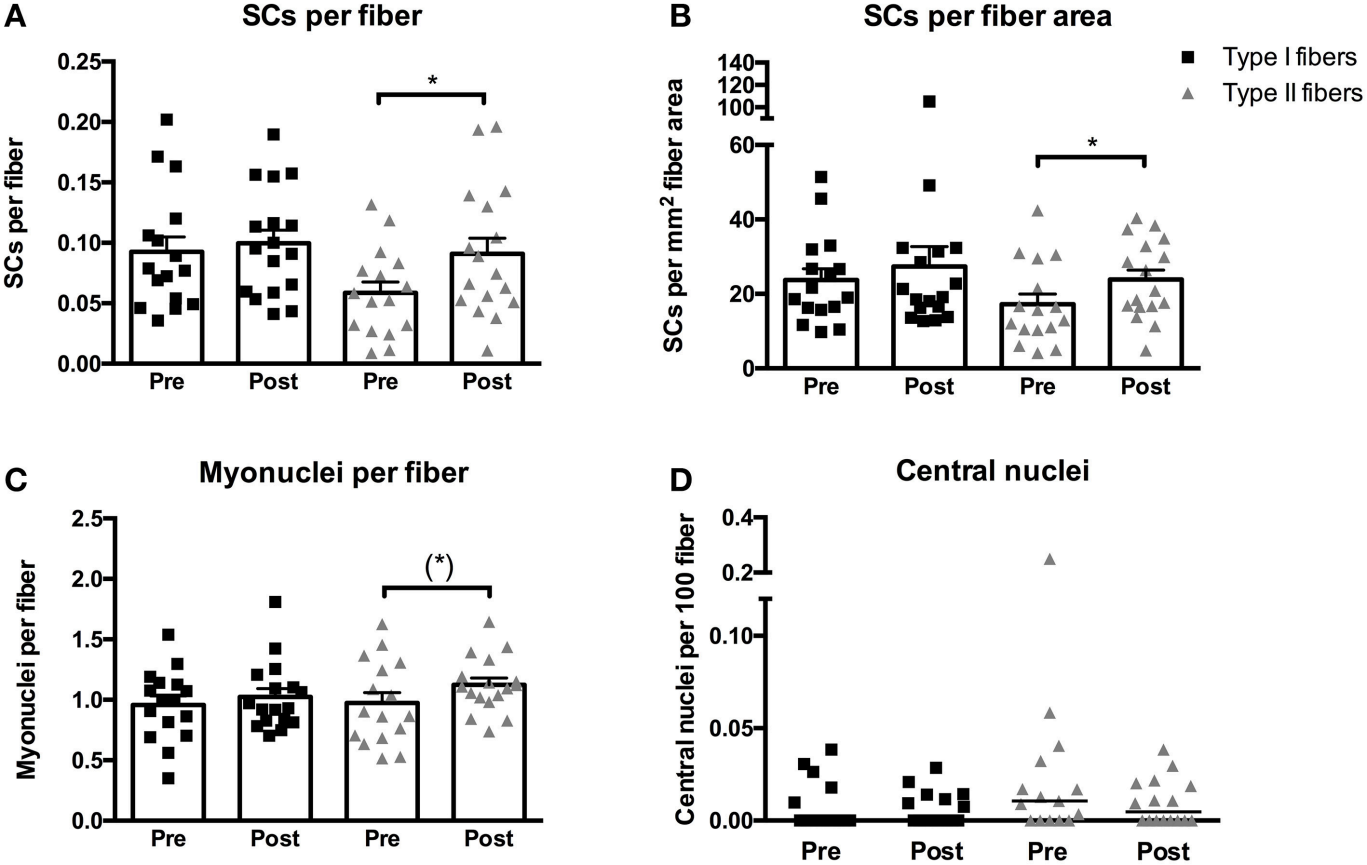
**Satellite cells (SCs) associated with type I or II fibers expressed per fiber (A) or per mm^**2**^ fiber area (B), myonuclei per fiber (C), and central nuclei per fiber (D) evaluated from biopsies obtained pre- and post-training in multiple sclerosis patients**. Data are presented as individual values and mean ± SEM **(A–C)** or individual data-points and median bar **(D)**. Significant differences from pre to post are denoted by ^*^p < 0.05 and tendencies by (^*^) *p* = 0.06.

The training intervention in MS patients elicited an increase in SCs per fiber associated with type II fibers of 165 ± 68% (*p* < 0.05, Figure 3A). In contrast no changes were observed in SCs per fiber in type I fibers. Similarly, an increase of 135 ± 63% was observed when expressed as SCs per mm^2^ fiber area in type II fibers (*p* < 0.05, Figure 3B), whereas no changes were found in SCs per mm^2^ fiber area in type I fibers.

The myonuclear content of type II fibers displayed a tendency toward an increase of 35 ± 14% following training (*p* = 0.06, Figure 3C), whereas no changes were observed in type I fibers. No changes were observed in the size of the myonuclear domain and the difference between the myonuclear domain size of type I (4348 ± 402 μm^2^ × myonuclei^−1^) and type II (3683 ± 399 μm^2^ × myonuclei^−1^) fibers persisted following training (*p* < 0.05).

No changes were observed in the number of central nuclei of either type I or type II fibers (Figure 3D). In addition, no differences were observed between type I and type II fibers at post-training.

### Skeletal muscle fibrosis and lipid content; effect of MS and training

**Figure 4:**
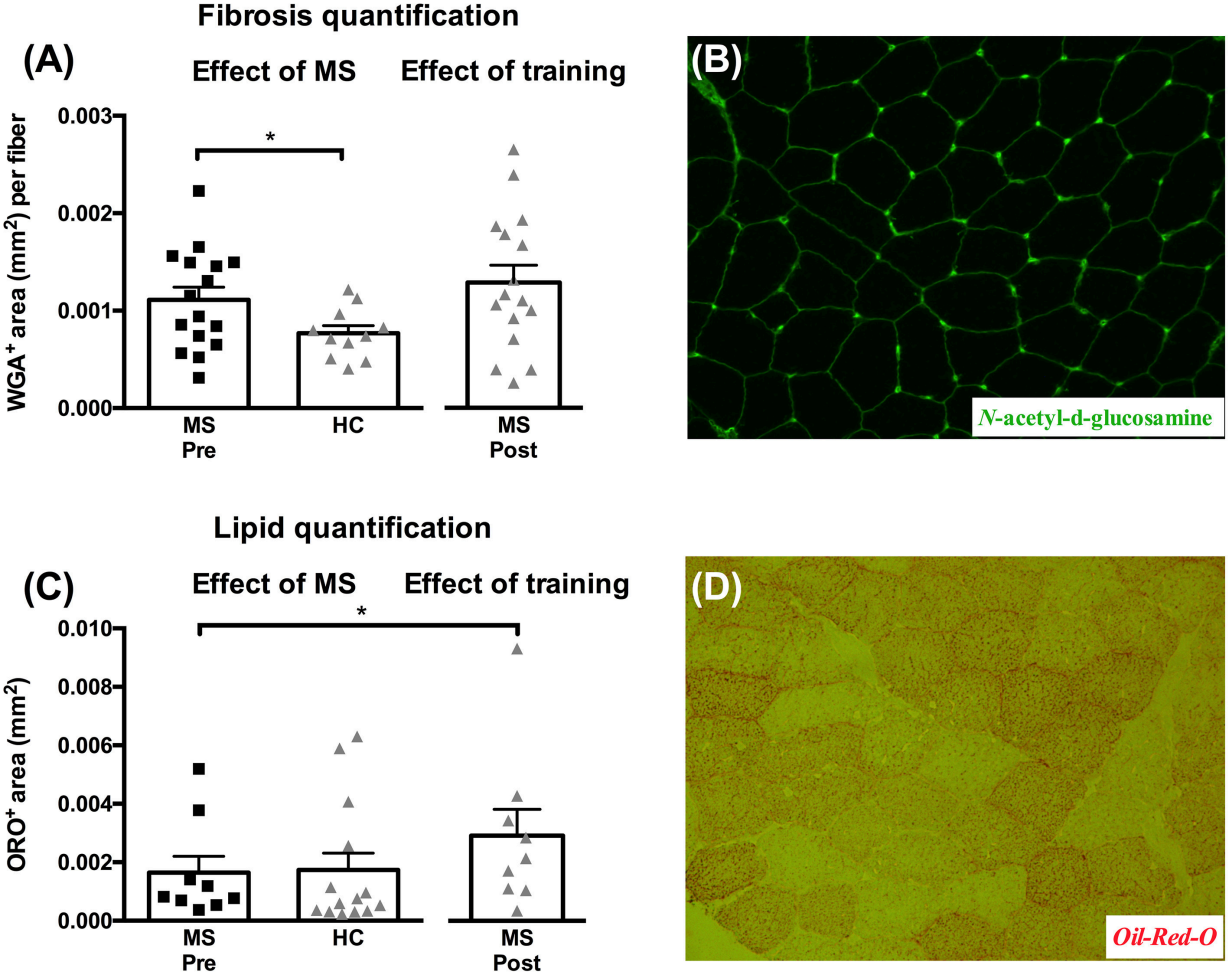
**Muscle tissue area (mm^**2**^) occupied by connective tissue/fibrosis per fiber (A) and lipid content (C) evaluated from biopsies obtained from healthy controls (HC), and multiple sclerosis patients (MS) pre- and post-training**. Data are presented as individual values and mean ± SEM. Significant differences from pre to post are denoted by ^*^p < 0.05. Representative images of connective tissue stain (*N*-acetyl-d-glucosamine stain) and lipids (Oil-Red-O) area shown in **(B)** and **(D)**, respectively.

At baseline we observed a difference in muscle tissue fibrosis (Figure 4A, *p* = 0.05) between MS (0.0011 ± 0.00013 mm^2^/fiber) and HC (0.00077 ± 0.000078 mm^2^/fiber), whereas no differences were detected from pre- to post-training in MS. As for lipid content we found no differences at baseline between MS and HC, whereas we did detect a significant increase post-training in MS of 117 ± 37% (Figure 4C, *p* < 0.05). Representative images for *N*-acetyl-d-glucosamine and Oil-Red-O staining's are shown in Figures 4B,D.

### Association between fiber size, satellite cell, and myonuclear content

**Figure 5:**
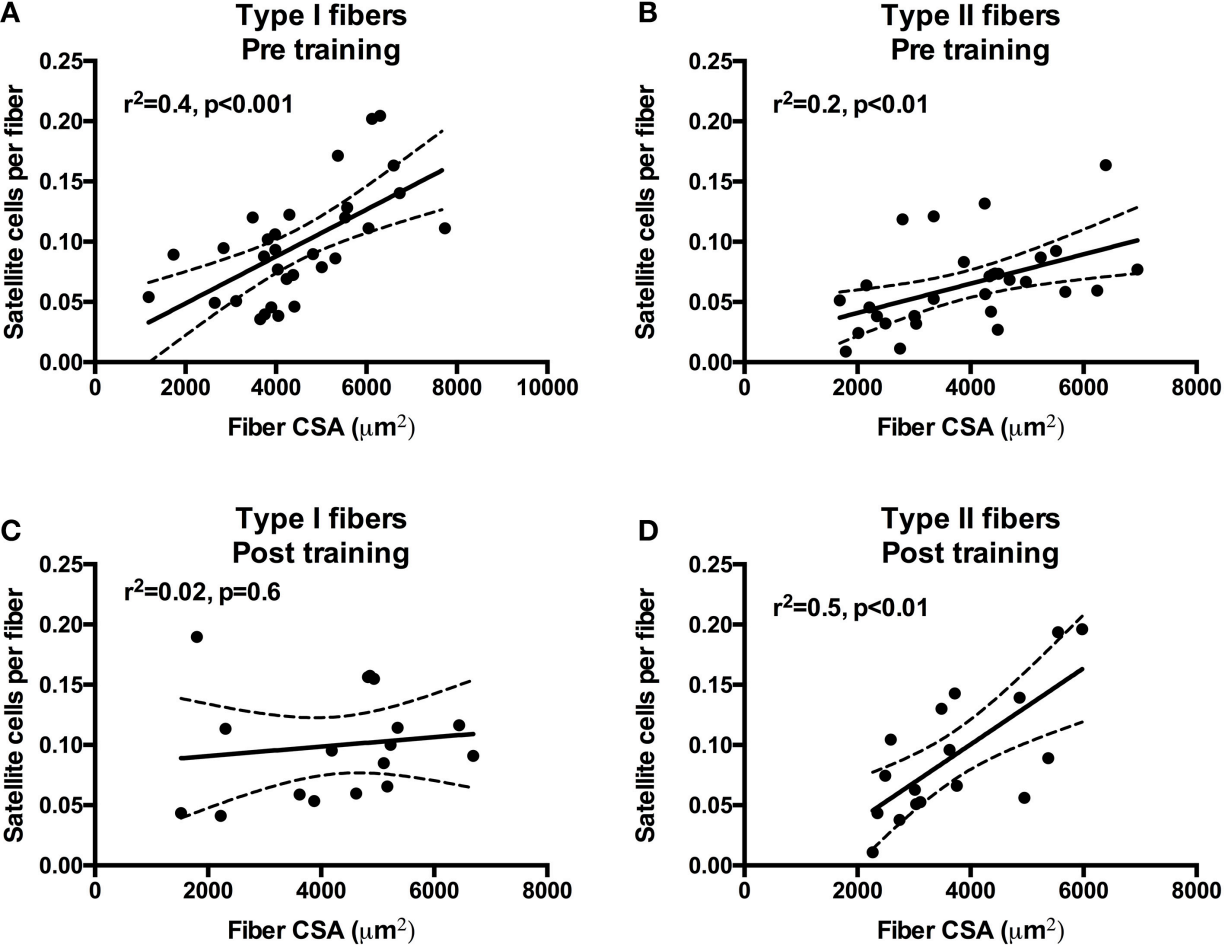
**Correlations and scatter plots of type I and type II fiber cross-sectional area (CSA) and satellite cell content at pre-training (A,B) and post-training (C,D)**. Dashed lines denote the 95% confidence intervals. Pearson correlation coefficients (*r*^2^) and significance are shown for each correlation.

To investigate the association between fiber CSA and SC content we performed a correlation analysis on pre- as well as post-training data. At pre-training (both MS and HC included) the fiber CSA of type I and type II fibers was positively associated with SC content (Figures 5A,B). Conversely, at post-training only type II fCSA was positively associated SC content (Figures 5C,D). Notably, in type II fibers the *r*^2^ increased from 0.2 at pre-training to 0.5 at post-training. Furthermore, we assessed whether SC or myonuclei content at baseline was associated with the increase in CSA for type I or type II fibers. We observed a tendency for myonuclei content in type II fibers at baseline to be associated with type II fiber growth (*r*^2^ = 0.31, *p* = 0.06), whereas no other significant associations were observed (data not shown). Finally, we observed no associations between the increase in SC content, the accretion of myonuclei, and the myofiber growth (data not shown).

## Discussion

The skeletal muscle stem cells (i.e., SCs) are instrumental for muscle regenerative capacity, health, and function. In consequence, a thorough understanding of SC regulation and function in neurodegenerative disorders such as MS, may add important knowledge for the preservation of skeletal muscle mass and quality in MS, and in neurodegenerative disorders in general. The main findings of the present study is that (1) a lower SC content in type II compared to type I fibers is observed in MS as well as in healthy age-matched controls, (2) the decreased type II fiber size in MS is associated with a trend toward a lower myonuclei content and increased tissue fibrosis, and (3) combined resistance and cardiovascular training can increase the SC and myonuclei content in type II fibers.

### Satellite cells and myonuclei in healthy controls vs. MS

In the present study we observed a lower SC content per fiber in type II compared to type I fibers both in MS and healthy controls. The content of SCs per fiber is known to decline during aging in a fiber type specific manner (Verdijk et al., 2009a, 2013; McKay et al., 2012; Mackey et al., 2013); i.e., with type II fibers selectively demonstrating a lower number of SCs, even when normalizing for fiber size (Verdijk et al., 2009a; Mackey et al., 2013). It is not yet well-defined at which time-point during aging this fiber type difference becomes detectable, however, earlier studies have reported fiber type differences in SC content in both elderly (i.e., age 50–69) and senescent (i.e., age >70) subjects (Verdijk et al., 2013). The healthy controls and MS patients in the present study were slightly younger (i.e., mean age of 47.5 and 45.7, respectively) and our findings thus indicate that aging or inactivity *per se* may explain the lower SC content per fiber in type II fibers, while no significant effect of MS *per se* was observed. The effect of chronic disease on SC content *in vivo* in humans is only sparsely described (Snijders et al., 2011; Beenakker et al., 2012; Molsted et al., 2015) and none of these have included a healthy age-matched control group. Thus, while both type II diabetic patients (Snijders et al., 2011) and dialysis patients (Molsted et al., 2015) were reported to have a reduced SC content per fiber in type II compared to type I fibers, it is unclear if these fiber type differences were related to the disease *per se* and/or aging. Since both studies reported a mean patient age >50 years, the reported fiber type difference may in fact predominantly be related to aging (Verdijk et al., 2013) rather that to the specific pathological condition, as our data on MS vs. healthy controls indicate.

In addition to aging, the decline in type II fiber SCs may partially be explained by inactivity. In a recent study master athletes (i.e., athletes obtaining a major exercise volume throughout life) were observed to maintain a similar content of SCs in type I and type II fibers when normalizing to fiber area (Mackey et al., 2013). Furthermore, inactive young (i.e., mean age 24) as well as inactive elderly (i.e., mean age 66) were reported to have a lower SC content in type II compared to type I fibers when normalized to fiber area, suggesting that inactivity *per se* may drive the lower SC in type II fibers, irrespective of aging (Mackey et al., 2013). Interestingly, in the present study we observed a reduction in SCs per fiber area in type II compared to type I fibers in MS patients whereas no differences were detected in healthy controls, although no significant group difference between MS and healthy controls could be detected. As previously reported, MS patients display a lower type II fiber area compared to healthy controls (Wens et al., 2014) matched for age and BMI and with no questionnaire reported differences in activity levels (data not shown). Therefore, the lower type II fiber SC content when normalized to fiber size may be related to MS rather than aging or inactivity. In addition, we observed a greater degree of connective tissue/fibrosis (as evaluated by the *N*-acetyl-d-glucosamine staining) in MS patients compared to controls, whereas no differences were observed in lipid content. The mechanisms underlying these findings cannot be elucidated from the present study, however, earlier studies have reported MS to be associated with a greater level of systemic pro-inflammatory cytokines such as tumor necrosis factors alpha (TNFα) compared to healthy controls (Martins et al., 2011). When TNFα, which during normal muscle regeneration is transiently released from classically activated (pro-inflammatory) macrophages (Arnold et al., 2007), is chronically secreted and elevated this may hamper SC self-renewal through epigenetic repression of Pax7 expression (Palacios et al., 2010). Conversely, TNFα has recently been suggested to prevent muscle tissue fibrosis through induction of fibro-adipogenic progenitor apoptosis (Lemos et al., 2015) whereas TGFβ1 increased fibro-adipogenic progenitor differentiation into extra-cellular matrix producing cells. The increased connective tissue/fibrosis in MS vs. controls may therefore indicate decreased levels of TNFα/increased levels of TGFβ1, or at least a dysfunctional regulation of these cytokines compared to healthy controls. However, in the present study we did not measure TNFα or TGFβ1 and furthermore our speculation will require more studies with greater statistical power before any inferences can be suggested.

In regards to the previously reported atrophy of type II fibers in MS patients (Wens et al., 2014), we also observed a trend toward a lower content of myonuclei specifically in type II fibers in MS patients. Thus, the myofiber atrophy may be associated with a lower myonuclear content, which would serve to maintain the same myonuclear domain size (fCSA/myonuclei). Moreover, we did also observe a significant association between fCSA and SC content in both fiber types at baseline, suggesting that fiber size may partly explain the SC content or vice versa.

### Effect of training on satellite cell content and myonuclei accretion in MS patients

Following combined cardio vascular and resistance training the SC content per fiber as well as per fCSA increased in MS patients along with an increase in the myonuclear content of type II fibers. Resistance training has previously been shown to increase fCSA, SC, and myonuclear content in both young and old subjects (Kadi et al., 2004; Petrella et al., 2006; Verdijk et al., 2009a; Mackey et al., 2010; Farup et al., 2014). In elderly subjects the selective type II fiber atrophy appears to be partly reversible with resistance training (Kryger and Andersen, 2007; Verdijk et al., 2009a,b) which is associated with a selective increase in SCs in type II fibers (Verdijk et al., 2009a). Interestingly, high intensity endurance training can also increase the SC content of type II fibers and increase the content of SCs expressing the myogenic differentiation factor; MyoD (Verney et al., 2008; Hoedt et al., 2015). Although endurance training may induce minor increases in myofiber size, these studies indicate that SC content and activity can increase without significant changes in myofiber size (Verney et al., 2008; Joanisse et al., 2013; Hoedt et al., 2015). Consequently, the increase in SC content in response to exercise is not necessarily related to myofiber hypertrophy, although previous studies have indicated that pre-training SC content may predict myofiber hypertrophy potential (Petrella et al., 2006, 2008). We did observe a positive association between myonuclei content in type II fibers at baseline and the type II fiber hypertrophy observed post-training, however, we found no association between SC content at baseline and myofiber growth in contrast to previous studies (Petrella et al., 2008). Since the MS patients in the present study performed both endurance and resistance exercise this may confound these correlation analyses, as both can influence SC content, whereas resistance exercise is the primary inducer of myofiber growth.

Very few studies have, to our knowledge, investigated the effect of resistance training on SC content in patients suffering from chronic diseases. In patients undergoing dialysis no changes were reported in type II fiber SC content following 16 weeks of resistance training (Molsted et al., 2015). However, in the latter study the authors did report an increase in the myonuclear content of type II fibers suggesting that SCs had proliferated and committed to the myogenic differentiation program at an earlier time-point (Molsted et al., 2015). In support, we observed an increase in the content of myonuclei in type II fibers, which was accompanied by an increase in the SC content of type II fibers. Of note, the increase in type II fiber SC resulted in an elimination of the fiber type difference observed at baseline between type I and type II fibers, in accordance with earlier results on resistance training in elderly subjects (Verdijk et al., 2009a). We did not observe any changes in connective tissue/fibrosis content; however, we did find an increased in lipid (Oil-Red-O stain). This is most likely explained by increased lipid storage in the myofibers (intracellular lipids) in response to training, as observed in endurance athletes (Goodpaster et al., 2001; Amati et al., 2011), in contrast to extracellular lipid (e.g., adipocytes).

### Clinical implications

The present findings suggest that the SC pool may decrease with age or inactivity whereas any direct effect of MS is less clear. Interestingly, high intensity training in MS patients can, in part, reverse this age, inactivity or MS related decline and help preserve muscle mechanical and metabolic function. Importantly, our data indicate that the SCs are able to both proliferate and differentiate to support the accretion of myonuclei and thereby providing additional transcriptional capacity to assist the type II fiber growth. Collectively, this suggests an intact functionality of the SCs in MS patients. Moreover, the preserved function of the SC pool suggests that MS patients have maintained their ability to support muscle hypertrophy (as observed in the present study), but potentially also the regenerative capacity when experiencing a more severe muscle injury or insult. Immobilization for shorter or longer periods is often experienced during aging in general, but also during neurodegenerative diseases. Following immobilization induced atrophy, the inability of the SC pool to proliferate is associated with impaired regrowth in elderly subjects (Suetta et al., 2013), suggesting that the preserved function of the SCs in MS is important for the ability to recover following immobilization. Collectively, combined cardio vascular and resistance training provide a feasible strategy to increase type II fiber size while also increasing the muscle stem cell pool, which combined may support MS patients during recovery from muscle trauma, immobilization or simply deconditioning. However, when interpreting the present study it should be kept in mind that the sample size of the study is somewhat limited.

## Conclusion

In conclusion, we report that MS patients have a lower SC content in type II compared to type I fibers, however, this reduction was also observed in a healthy age matched control group. Furthermore, high intensity training reversed the reduction in type II fiber SC and induces differentiation of SC to provide additional myonuclei to the post-mitotic myofibers. Collectively, the SC pool in MS patients appear to maintain proliferative and differentiation capacity, which can be stimulated through high intensity training.

## Author contributions

Conception and design of research; IW, UD, BE conducted clinical trial; IW, CK performed experiments; JF, IW analyzed data; JF, IW interpreted results of experiments; JF, UD, IW drafted manuscript; JF, UD, IW edited and revised manuscript; JF, UD, CK, BE, IW approved final version of manuscript; JF, UD, CK, BE, IW.

## Funding

This work was supported by MS Fund, Limburg, Flanders, Belgium and the Danish Council for Independent Research/Medical Sciences, 5053-00195B.

### Conflict of interest statement

The authors declare that the research was conducted in the absence of any commercial or financial relationships that could be construed as a potential conflict of interest.
